# Ester Hydrolysis Differentially Reduces Aconitine-Induced Anti-hypersensitivity and Acute Neurotoxicity: Involvement of Spinal Microglial Dynorphin Expression and Implications for *Aconitum* Processing

**DOI:** 10.3389/fphar.2016.00367

**Published:** 2016-10-05

**Authors:** Teng-Fei Li, Nian Gong, Yong-Xiang Wang

**Affiliations:** King’s Lab, Shanghai Jiao Tong University School of PharmacyShanghai, China

**Keywords:** aconitine, benzolyaconine, aconine, anti-hypersensitivity, dynorphin A, neurotoxicity

## Abstract

Aconitines, including bulleyaconitine A, probably the most bioactive and abundant alkaloids in *Aconitum* plant, are a group of diester C19-diterpenoid alkaloids with one acetylester group attached to C8 of the diterpenoid skeleton and one benzoylester group to C14. Hydrolysis of both groups is involved in the processing of *Aconitum*, a traditional Chinese medicinal approach. We recently demonstrated that bulleyaconitine A produced anti-hypersensitivity, which was mediated by stimulation of spinal microglial dynorphin A expression. This study aimed to elucidate whether the acetylester and benzoylester groups are involved in aconitine-induced dynorphin A expression, anti-hypersensitivity, neurotoxicity in neuropathic rats. Intrathecal administration of aconitine and benzoylaconine (but not aconine) attenuated mechanical allodynia and heat hyperalgesia, with normalized ED_50_ values of 35 pmol and 3.6 nmol, respectively. Aconitine and benzoylaconine anti-allodynia was completely blocked by the microglial inhibitor, dynorphin A antiserum, and κ-opioid receptor antagonist. Aconitine and benzoylaconine, but not aconine, stimulated dynorphin A expression in cultured primary spinal microglia, with EC_50_ values of 32 nM and 3 μM, respectively. Intrathecal aconitine, benzoylaconine and aconine induced flaccid paralysis and death, with normalized TD_50_ values of 0.5 nmol, 0.2 μmol, and 1.6 μmol, respectively. The TD_50_/ED_50_ ratios of aconitine and benzolyaconine were 14:1 and 56:1. Our results suggest that both the C8-acetyl and C14-benzoyl groups are essential for aconitine to stimulate spinal microglial dynorphin A expression and subsequent anti-hypersensitivity, which can be separated from neurotoxicity, because both benzoylaconine and aconine differentially produced anti-hypersensitivity and neurotoxicity due to their different stimulatory ability on dynorphin A expression. Our results support the scientific rationale for *Aconitum* processing, but caution should be taken to avoid overprocessing and excess hydrolysis of benzolyaconine to aconine.

## Introduction

***A****conitum* is widely prescribed in China and other Eastern Asia countries for its therapeutic efficacy against nociception, inflammation, rheumatism, and cardiologic and neurologic disorders (Chen and Liang, 1982; Hikino et al., 1983; Chen et al., 2008). Approximately 170 alkaloids have been identified in *Aconitum* and classified into four categories: *bis*-, C20-, C19-, and C18-diterpenoid alkaloids (Ameri, 1998; Chodoeva et al., 2013). Aconitines, including aconitine, mesaconitine, hypaconitine, bulleyaconitine A, yunaconitine, and 3-acetylaconitine, are probably the most abundant and bioactive C19-diester diterpenoid alkaloids identified in *Aconitum*. Aconitine, with two hydroxyl groups at the C3 and C15 of the skeleton, shares a very similar structure to bulleyaconitine A (Li et al., 2016). For C19-diester diterpenoid alkaloids, one acetylester group is attached to the C8 while another benzoylester group is attached to the C14. It is generally accepted that both the C-8 acetylester and C-14 benzoylester are required for aconitines to produce antinociceptive and neurotoxic effects (Friese et al., 1997; Gutser et al., 1998; Bello-Ramirez and Nava-Ocampo, 2004a,b). It has been claimed that the interactions with neuronal sodium channels were responsible for the analgesic and neurotoxic effects of aconitines (Ameri, 1998; Gutser et al., 1998; Wang et al., 2007; Wang et al., 2008; Chan, 2009; Chodoeva et al., 2013). By quantitative structure-activity relationship analysis, a significant linear relationship was established between LD_50_ and the antinociceptive ED_50_ of 12 diterpenoid alkaloids, and it was hypothesized that the analgesic effects of these alkaloids were secondary to their toxic effects (Bello-Ramirez and Nava-Ocampo, 2004a), although there has been no evidence for a causal relationship between them.

However, we recently discovered that bulleyaconitine A anti-hypersensitivity can be separated from its neurotoxicity by the mechanism of action. The microglial inhibitor minocycline (Raghavendra et al., 2003; Hua et al., 2005) completely abolished the antinociceptive effects of bulleyaconitine A (but not dynorphin A or the sodium channel blocker bupivacaine) in neuropathic rats, but did not significantly affect bulleyaconitine A neurotoxicity. Treatment with bulleyaconitine A significantly increased the dynorphin A level (both the gene expression and peptide content) in the spinal cord and cultured primary spinal microglia (but not astrocytes or neurons) from neonatal and adult rats, which was completely attenuated by minocycline. In contrast, the sodium channel blockers bupivacaine and lidocaine did not stimulate dynorphin A expression or inhibit the stimulatory effects of bulleyaconitine A on the dynorphin A expression in spinal microglia (Li et al., 2016). It is not known whether the C8-acetylester and C14-benzoylester groups are responsible for aconitine’s stimulation on spinal microglial dynorphin A expression and subsequent anti-hypersensitivity in neuropathic pain.

**FIGURE 1:**
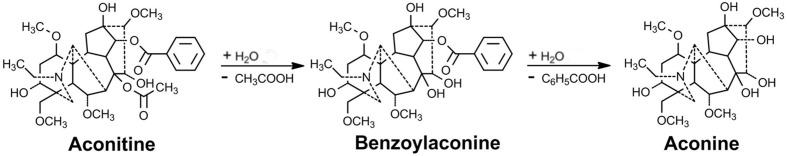
**Illustration of the aconitine detoxification process by the two-step ester hydrolysis**.

As a foremost highly toxic plant with marked effects on the central nervous and cardiovascular systems, *Aconitum* exhibits a narrow therapeutic window, which brings challenges to safe use as a medicine. From a standard approach of traditional Chinese medicine, *Aconitum* must be generally processed by heating in boiling or steaming water, or treating with a dilute acid or alkali solution for a few hours, to reduce the toxicity before it can be used in medicine or in the herbal commercial market. It has been demonstrated that the processing of *Aconitum* dramatically deacetylates diester aconitines (such as aconitine, mesaconitine, and hypaconitine) at the C8 to form monoester diterpene alkaloid benzoylaconines, for instance, benzoylaconine, benzoylmesaconine, and benzoylhypaconine. These monoesters are further debenzoylated at the C14 to form hydramine-type aconines, such as aconine, mesaconine, and hypaconine (Gozuacik et al., 2003; Singhuber et al., 2009; Sun et al., 2009; Lu et al., 2010). The two-step processing pathway of the diester diterpenoid alkaloids represented by aconitine is presented in **Figure 1**. Interestingly, the same metabolic pathway as physical processing also takes place in the living body. The concentration of aconitine is decreased, whereas the concentrations of benzoylaconine and aconine are increased, in the blood and body organs including the liver and kidneys in mice (Wada et al., 2005), rats (Wang et al., 2006), and humans (Zhang et al., 2005). The metabolism of *Aconitum* alkaloids is mainly carried out by carboxylesterases (Zhang et al., 2015).

Although the antinociceptive properties of aconitines, from the perspective of the mechanism of action, can be separated from their toxicity (Li et al., 2016), their analgesic effects are closely associated with the toxicity and cannot be separated. This raises questions that whether the process makes scientific sense if the procedure does not differentially reduce the antinociceptive and toxic effects of *Aconitum* and its derived aconitines. It was reported that all three deacetylated metabolites, benzoylaconine, benzoylmesaconine, and benzoylhypaconine, inhibited acetic acid–induced writhing responses, with activities roughly 100-fold less than those of their parent compounds aconitine, mesaconitine, and hypaconitine (Hikino et al., 1979). It was also summarized from the literature that the LD_50_ values of aconitine, benzoylaconine, and aconine administered by intravenous injection in mice were 0.12, 23, and 120 mg/kg, respectively (Mizugaki and Ito, 2005). However, to our knowledge, no direct head-to-head comparative study has been performed to compare aconitines and their hydrolyzed products benzoylaconines and aconines on antinociception and neurotoxicity. It is important to address the issue, particularly when the new hypothesis of dynorphin A expression is in consideration.

Therefore, we aimed to study the mechanism that underlies the antinociceptive effects of aconitine and its hydrolyzed products benzoylaconine and aconine, and to elucidate their differential effects on spinal microglial dynorphin A expression, antinociception, and acute neurotoxicity. The experimental procedures included the determination of (1) the anti-hypersensitivity effects of aconitine, benzolyaconine, and aconine in a rat model of neuropathy induced by tight ligation of spinal nerves; (2) the blockade effects of the specific antiserum against dynorphin A, selective κ-opioid receptor antagonist, and microglial inhibitor on aconitine and benzolyaconine antinociception; (3) the stimulatory effects of aconitine, benzolyaconine, and aconine on dynorphin A expression in primary cultures of microglia originating from the spinal cord; (4) and the acute neurotoxicity of aconitine, benzoylaconine, and aconine in neuropathic rats. The dose-response analysis was particularly used in all tests for quantitative assessment of their differential effects.

## Materials and Methods

### Drugs and Reagents

Aconitine was obtained from Guokang Bio-Pharmaceutical (Baoji, China), and benzolyaconine and aconine were purchased from Nanjing Zelang Bio-Pharmaceutical (Nanjing, China). Their molecular masses were confirmed by in-house mass spectroscopy, and their purity was greater than 98% as determined by the manufacturers using high-performance liquid chromatography. Nor-binaltorphimine (nor-BNI) and minocycline were purchased from Abcam (Cambridge, U.K.) and Yuanye Biotech (Shanghai, China), respectively. The rabbit polyclonal antibody neutralizing dynorphin A was purchased from Phoenix Pharmaceuticals (Burlingame, CA, USA). Based on the manufacturer’s information, the dynorphin A antiserum was specific to dynorphin A (100%), but not to dynorphin B (0%), β-endorphin (0%), α–neo-endorphin (0%), or leu-enkephalin (0%). Its specificity was also validated by the antigen absorption test by other laboratories (Wakabayashi et al., 2010; Yamada et al., 2013).

### Experimental Animals

Male adult (200 ± 20 g body weight) and 1-day-old neonatal Wistar rats were obtained from the Shanghai Experimental Animal Institute for Biological Sciences (Shanghai, China). The rats were caged in a group of four with thick sawdust bedding at standard room temperature, under a 12-h/12-h reversed light-dark cycle (7:00 AM to 7:00 PM) at a constant temperature of 22°C ± 2°C. The adult rats received food and water *ad libitum* and were acclimatized to the laboratory environment for 3–5 days before the experiments. The experimental study groups (six rats per group except for the neurotoxicity study, which had 10 rats per group) were randomly assigned, and the researcher was blind to the results of the behavior tests. The research protocols were approved by the Animal Care and Welfare Committee of Shanghai Jiao Tong University.

### Primary Microglial Cell Culture

Glial cells were isolated from the 1-day-old neonatal rats. The isolated spinal cords were minced and then incubated with trypsin. Dissociated cells were suspended in 75-cm^2^ tissue culture flasks (1 × 10^7^ cells/flask) precoated with poly-L-lysine and maintained in a 5% CO_2_ incubator at 37°C. After culture for 10 days, the microglial cells were prepared as floating cell suspensions by shaking the flask at 260 rpm for 2 h. The aliquots were transferred to plates, and the unattached cells were removed by washing with serum-free Dulbecco’s modified Eagle’s medium. The harvested microglia exhibited purity greater than 95% as determined by CD11b (OX42) immunoreactivity.

### RNA Extraction, Reverse Transcription, and Real-Time Quantitative Polymerase Chain Reaction

RNA extraction, reverse transcription, and real-time quantitative PCR were performed as previously described (Chen et al., 2012). The spinal lumbar enlargements of rats were collected and mechanically homogenized with an electronic microhomogenizer at 4,000 rpm for 10 s in TRIzol on ice. Total RNA from the spinal lumbar enlargements were isolated using TRIzol reagent (Invitrogen, Grand Island, NY, USA). A 1-μg sample of total RNA was reverse-transcribed with a ReverTra Ace qPCR RT-Kit (Toyobo, Japan). Real-time quantitative PCR was carried out with the Mastercycler ep realplex (Eppendorf, Germany) using the RealmasterMix (SYBR Green I) (Toyobo). The fold change was calculated with the 2^-ΔΔCt^ method after normalization to the gene of *gapdh*. The primers used were: 5′-CCA AGG TCA TCC ATG ACA AC-3′ (gapdh forward), 5′-TCC ACA GTC TTC TGA GTG GC-3′ (gapdh reverse) (Gong et al., 2014), 5′-ACT GCC TGT CCT TGT GTT CC-3′ (prodynorphin forward), and 5′-CCA AAG CAA CCT CAT TCT CC-3′ (prodynorphin reverse) (Leitl et al., 2014).

### Intrathecal Catheterization and Injection in Rats

An 18-cm polyethylene catheter (PE-10: 0.28-mm inner diameter and 0.61-mm outer diameter, Clay Adams, Parsippany, NJ, USA) with a volume of 13 μL was inserted into the lumbar level of the spinal cord as described elsewhere under inhalational isoflurane anesthesia (4% for induction and 1% for maintenance) run by an anesthesia meter (Ugo Basile Gas Anesthesia System, Comerio, Italy). Two days after recovery from anesthesia, the placement of the catheter in the spinal cord was verified by administration of 4% lidocaine (10 μL, followed by 15 μL of saline for flushing). Rats that had no motor impairment after placement of the intrathecal catheter were considered for the study, and rats that developed immediate bilateral paralysis of the hindlimbs after intrathecal administration of lidocaine were selected for the study. For intrathecal administration of the control and test articles, 10 μL of each drug was injected with a 50-μL microinjector (Shanghai Anting Micro-Injector Factory, Shanghai, China) and was flushed with 15 μL of normal saline solution.

### Rat Model of Neuropathic Pain

To induce hypersensitivity to neuropathic pain, the rats were subjected to spinal nerve ligation as previously described Kim and Chung (1992) and Zhang et al. (2013). Unilateral ligation of the L5 and L6 spinal nerves was performed under inhalational isoflurane anesthesia (4% for induction and 1% for maintenance) run by an anesthesia meter (Ugo Basile Gas Anesthesia System). The left L5 and L6 spinal nerves were isolated and tightly ligated with 6-0 silk thread. After ligation, the wound was sutured and the rats were returned to their home cages for recovery. After spinal nerve ligation, only those rats with marked unilateral allodynia to mechanical stimulation (i.e., a hindpaw withdrawal threshold of less than 8 g on the operated side) and with no major impairment were included in the study. Drug testing started 2–4 weeks after spinal nerve ligation.

### Behavioral Assessments of Mechanical Allodynia and Heat Hyperalgesia in Rats

To evaluate mechanical allodynia, the animals were acclimatized for at least 30 min to the test environment, namely, a plexiglass box on a metal grid (0.5 cm × 0.5 cm). The hindpaw withdrawal threshold was measured with a 2450 CE Electronic von Frey hair (IITC Life Science Inc, Woodland Hill, CA, USA). An electronic handheld transducer with a No. 15 monofilament was applied perpendicularly to the medial surface of the hindpaws with increasing force (ranging from 0.1 to 90 g) until the rat suddenly withdrew or licked the hindpaw. The lowest force that produced a withdrawal response was considered to be the threshold, which was the mean of three repeated measurements made in a 5-min interval.

To assess heat hyperalgesia, the rats were put in a plexiglass box on an elevated glass surface. After an adaptation period of at least 30 min, radiant heat was applied to the plantar medial surface of each hindpaw. The hindpaw withdrawal latency was measured with a Model 390G Plantar Test Analgesia Meter (IITC Life Science Inc.). To prevent tissue damage, the latency cutoff was set at 30 s. The paw withdrawal latency was defined as the time from the onset of radiant heat application to the withdrawal of the hindpaw. Both hindpaws were tested independently three times with a 5-min interval between trials. The result for each test was calculated as the mean of the three repeated measurements.

### Rat Acute Neurotoxicity

The rats were observed for acute neurotoxicity after each intrathecal injection of test articles, with a focus on motor blockade effects and survival. Survival and abnormal motor-blockade behaviors, including asthma, abdominal breathing, locomotion difficulty, and paralysis, were continuously monitored for 24 h after the treatment, with special attention given during the first 4 h. Paralysis was defined as an inability to negotiate a 60-degree inclined plane (Durant and Yaksh, 1986). Death was defined as respiratory arrest over a period of 5 min. The paralysis and mortality rates and their latencies were quantitatively recorded and tabulated.

### Statistical Analysis

The percentage of maximal possible effect (% MPE) was calculated using the following formula: (postdrug threshold in ipsilateral hindlimb – baseline threshold in ipsilateral hindlimb)/(baseline threshold in contralateral hindlimb – baseline threshold in ipsilateral hindlimb) × 100 (Bowersox et al., 1996). A % MPE value near 100 indicates normal mechanical thresholds (i.e., near contralateral thresholds), and a value near 0 indicates allodynia. For the dose-response curve analysis, the parameters (i.e., minimum effect; E_max_, median effective concentration or dose, or median toxic or lethal dose [EC_50_, ED_50_, TD_50_, or LD_50_], and Hill coefficient [n]) were calculated from the individual dose-response curves using the Prism program (version 5.01, GraphPad Software, San Diego, CA, USA). To determine the parameters of the dose-response curves, the values of response (Y) were fitted by non-linear least-squares curves to the relation Y = a + bx, where x = [D]^n^/(ED_50_^n^ + [D]^n^), [C]^n^/(EC_50_^n^ + [C]^n^), [D]^n^/(TD_50_^n^ + [D]^n^) or [D]^n^/(LD_50_^n^ + [D]^n^), to give each value of ED_50_, EC_50_, TD_50_, or LD_50_ and b (E_max_) yielding a minimum residual sum of squares of deviations from the theoretical curve (Wang and Pang, 1993).

The data are presented as the mean ± standard error of the mean (SEM) or with 95% confidence limits, and no data were missing. Statistical significance was evaluated by two-way repeated-measures ANOVA, which was followed by a *post hoc* Student–Newman–Keuls test when a statistically significant drug (dose) effect was observed with factors of drug (dose), time, and their interaction. Probability values were two-tailed, and the statistical significance criterion (*P*-value) was 0.05.

## Results

### Anti-hypersensitivity Effects of Aconitine, Benzolyaconine, and Aconine

**FIGURE 2:**
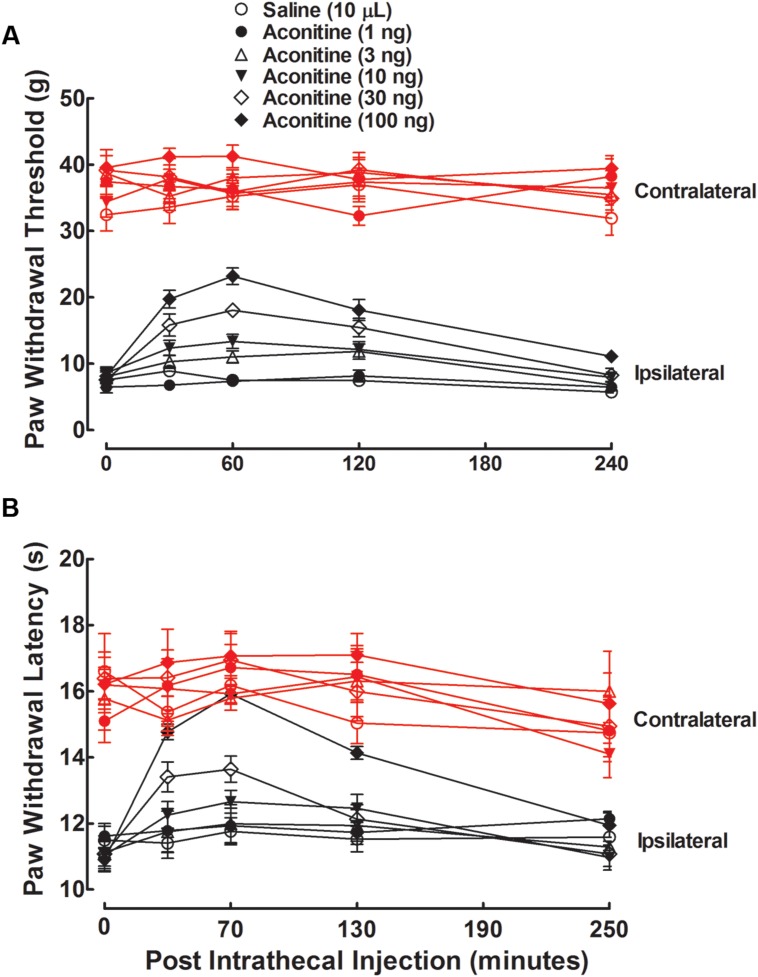
**Antinociceptive effects of intrathecal administration of aconitine on mechanical allodynia **(A)** and heat hyperalgesia **(B)** in spinal L5/L6 nerve–ligated neuropathic rats.** Rats received a single intrathecal administration of saline solution and aconitine (1, 3, 10, 30, and 100 ng). Withdrawal responses to mechanical and thermal stimuli in the contralateral and ipsilateral hindpaws were consecutively measured with 10-min intervals. Data are presented as the mean ± SEM (six per group).

The anti-hypersensitivity effects of aconitine, benzolyaconine, and aconine were studied in rats with neuropathy induced by spinal nerve ligation. Six groups of neuropathic rats received a single intrathecal bolus injection of normal saline solution (10 μL) or aconitine (1, 3, 10, 30, or 100 ng). The withdrawal thresholds to von frey monofilaments and withdrawal latency to radiant heat were consecutively measured (at an interval of 10 min) in both the contralateral and ipsilateral paws before and 30, 60, 120, and 240 min or 40, 70, 130, and 250 min after injection. As shown in **Figures 2A,B**, tight ligation of the L5 and L6 spinal nerves produced marked mechanical allodynia and heat hyperalgesia in the ipsilateral paws. The paw withdrawal responses remained unchanged during the 4-h observation period in rats treated with saline solution. Aconitine administered by intrathecal injection did not significantly alter the withdrawal responses in the contralateral paws, but it remarkably alleviated mechanical allodynia and heat hyperalgesia in the ipsilateral paws in a time-dependent manner, with a peak effect at 1 h after injection.

**FIGURE 3:**
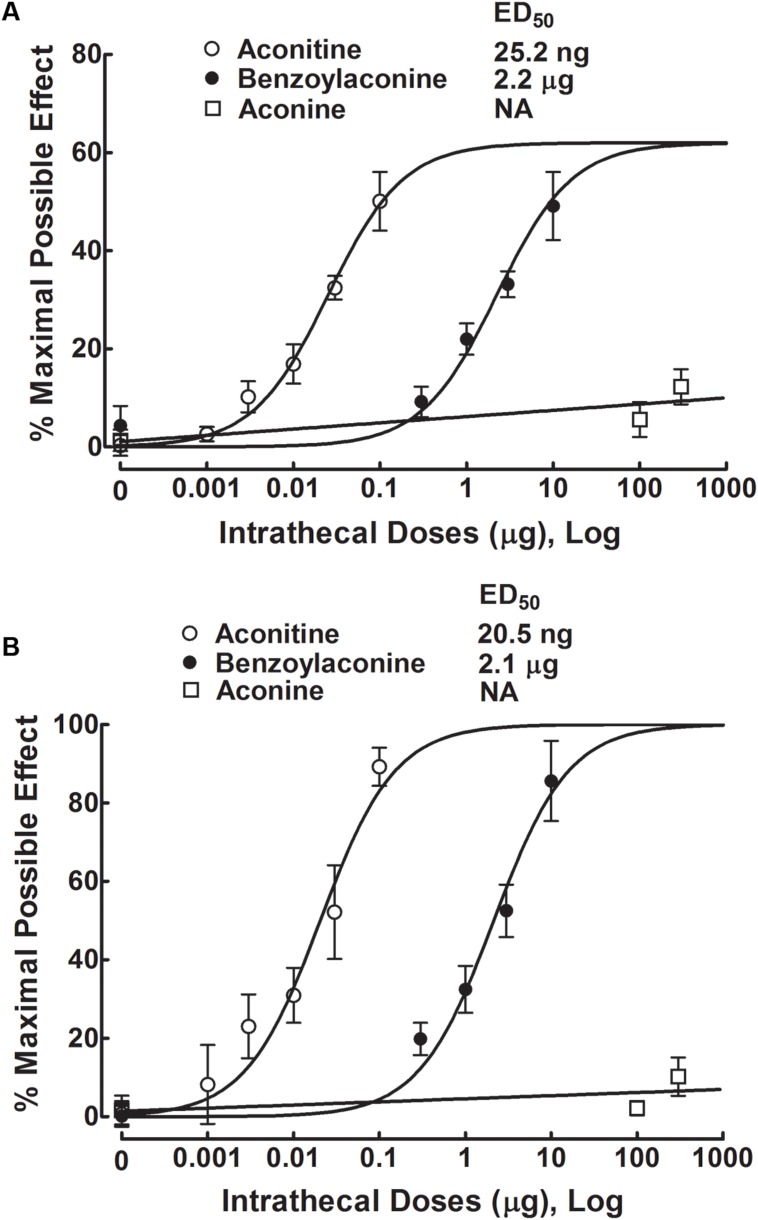
**Dose-inhibitory response curves of aconitine, benzolyaconine, and aconine on mechanical allodynia (**A**) and heat hyperalgesia (**B**) in spinal L5/L6 nerve–ligated neuropathic rats.** % Maximal possible effects of aconitine, benzolyaconine, and aconine were calculated 1 h after injection from original data of **Figures 2**, **4**, and **5**. The dose-response analyses were best projected by the non-linear least-squares method.

The anti-hypersensitivity effect of aconitine was dose-dependent. The % MPE values were calculated from each dose of aconitine at 1 h after injection, and the dose-response curves were projected. For the blockade of mechanical allodynia, the Emax and ED_50_ values were 61.9% MPE and 25.2 ng or 39.0 pmol (95% confidence limits: 18.8–33.8 ng or 29.1–52.3 pmol) (**Figure 3A**). For the blockade of thermal hyperalgesia, the Emax and ED_50_ values were 100% MPE and 20.5 ng or 31.7 pmol (95% confidence limits: 13.4–31.4 ng or 20.8–48.6 pmol) (**Figure 3B**).

**FIGURE 4:**
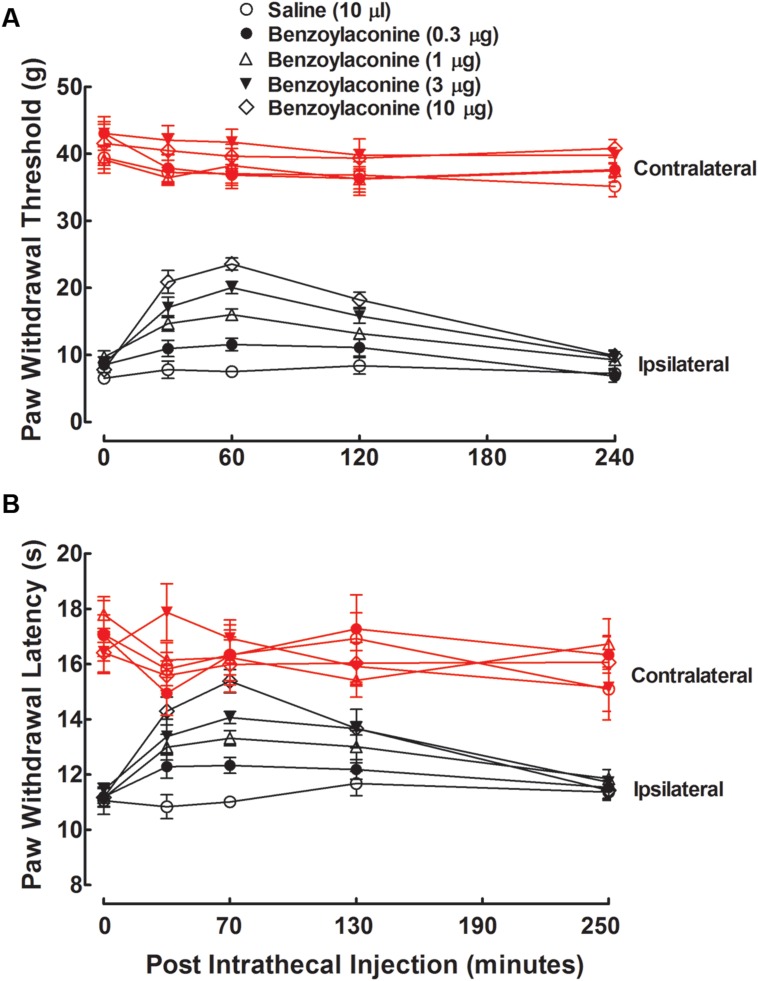
**Antinociceptive effects of intrathecal administration of benzolyaconine on mechanical allodynia **(A)** and heat hyperalgesia **(B)** in spinal L5/L6 nerve–ligated neuropathic rats.** Rats received a single intrathecal administration of saline solution and benzolyaconine (0.3, 1, 3, and 10 μg). Withdrawal responses to mechanical and thermal stimuli in the contralateral and ipsilateral hindpaws were consecutively measured with 10-min intervals. Data are presented as the mean ± SEM (six per group).

Five groups of neuropathic rats received a single intrathecal bolus injection of normal saline solution (10 μL) or benzolyaconine (0.3, 1, 3, or 10 μg). In the ipsilateral paws, intrathecal injection of benzolyaconine alleviated the mechanical allodynia and heat hyperalgesia in a dose- and time-dependent manner (*P* < 0.05 by two-way ANOVA). Benzolyaconine did not significantly affect the paw withdrawal responses in the contralateral paws (**Figures 4A,B**). The % MPE values were calculated from each dose of benzolyaconine at 1 h after injection, and the dose-response curves were projected. The Emax and ED_50_ values for anti-allodynia were 56.4% MPE and 2.2 μg or 3.6 nmol (95% confidence limits: 1.6–3.2 μg or 2.6–5.2 nmol) (**Figure 3A**). Accordingly, the Emax and ED_50_ values for anti-hyperalgesia were 100% MPE and 2.1 μg or 3.5 nmol (95% confidence limits: 1.5–3.0 μg or 2.5–5.0 nmol) (**Figure 3B**).

**FIGURE 5:**
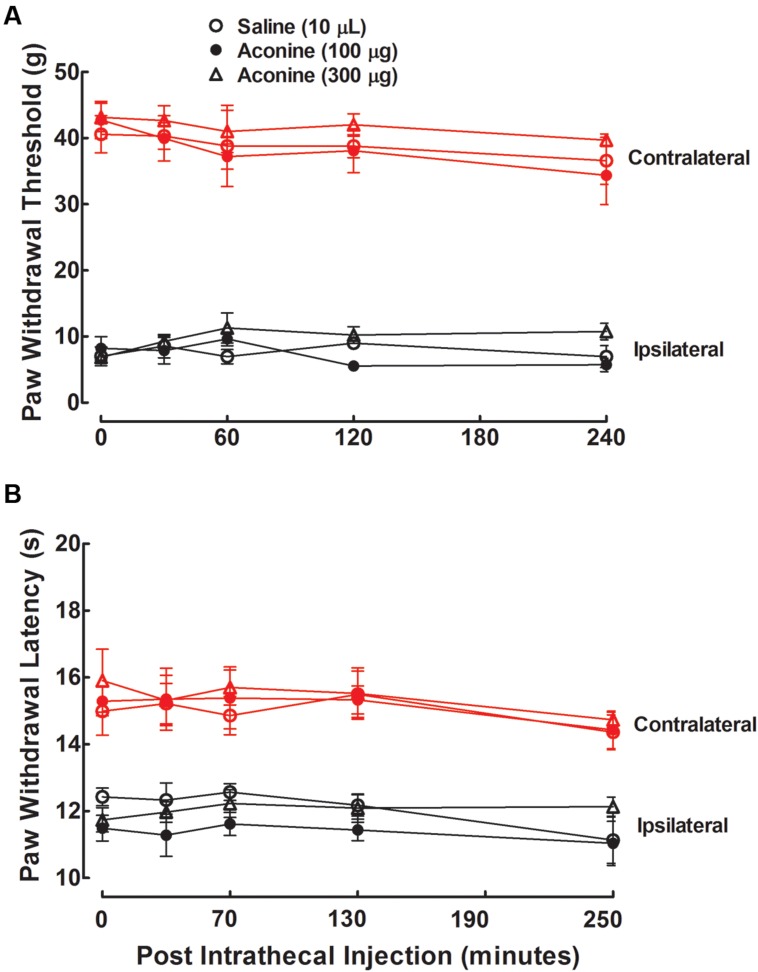
**Effects of intrathecal administration of aconine on mechanical allodynia **(A)** and heat hyperalgesia **(B)** in spinal L5/L6 nerve–ligated neuropathic rats.** Rats received a single intrathecal administration of saline solution and aconine (100 and 300 μg). Withdrawal responses to mechanical and thermal stimuli in the contralateral and ipsilateral hindpaws were consecutively measured with 10-min intervals. Data are presented as the mean ± SEM (six per group).

Three groups of neuropathic rats received a single intrathecal bolus injection of normal saline solution (10 μL) or aconine (100 or 300 μg). The highest dose of 300 μg was selected due to that a higher dose might lead to obvious motor-blockade behaviors or death. As shown in **Figures 5A,B**, intrathecal injection of aconine up to 300 μg did not produce significant anti-allodynia or anti-hyperalgesia in the ipsilateral paws, nor did it significantly affect the paw withdrawal responses in the contralateral paws. The % MPE of aconine at 1 h after injection are also presented in **Figures 3A,B** on mechanical allodynia and heat hyperalgesia.

**Table 1** summarizes the ED_50_ values in mole base for aconitine, benzolyaconine, and aconine to block pain hypersensitivity. Because anti-allodynia and anti-hyperalgesia were closely correlated, their ED_50_ values were averaged to obtain a normalized ED_50_ for anti-hypersensitivity. The ED_50_ ratios for aconitine and benzolyaconine to block mechanical allodynia and heat hyperalgesia were 1:92 and 1:110, with a normalized ED_50_ ratio of 1:102.

**Table 1:** The median effective dose (ED)_50_, toxic dose (TD)_50_ (for flaccid paralysis), and lethal (LD)_50_ and their normalized values, and the therapeutic index values for aconitine, benzolyaconine, and aconine given by intrathecal injection to produce anti-hypersensitivity and acute neurotoxicity in spinal nerve-ligated neuropathic rats (6 and 10 per group for the anti-hypersensitivity and neurotoxicity studies, respectively).

Compound	Anti-hypersensitivity (ED_50_)			Toxicity (TD_50_ or LD_50_)			Therapeutic Index		
			
	Allodynia	Hyperalgesia	Normalized	Paralysis	Mortality	Normalized	TD_50_/ED_50_	LD_50_/ED_50_	Normalized
Aconitine	39 pmol	32 pmol	35 pmol	0.4 nmol	0.6 nmol	0.5 nmol	10:1	19:1	14:1
Benzolyaconine	3.6 nmol	3.5 nmol	3.6 nmol	0.2 μmol	0.2 μmol	0.2 μmol	56:1	57:1	56:1
Aconine	NA	NA	NA	1.5 μmol	1.7 μmol	1.6 μmol	NA	NA	NA
Aconitine/Benzoyl-Aconine	1:92	1:110	1:102	1:452	1:421	1:400			
Benzolyaconine/Aconine	NA	NA	NA	1:8	1:7	1:8			
Aconitine/Aconine	NA	NA	NA	1:3,750	1:2,833	1:3,200			


*NA, not applicable.*

### Blockade Effects of the Dynorphin A Antiserum, Nor-BNI, and Minocycline on Aconitine and Benzolyaconine Anti-allodynia

**FIGURE 6:**
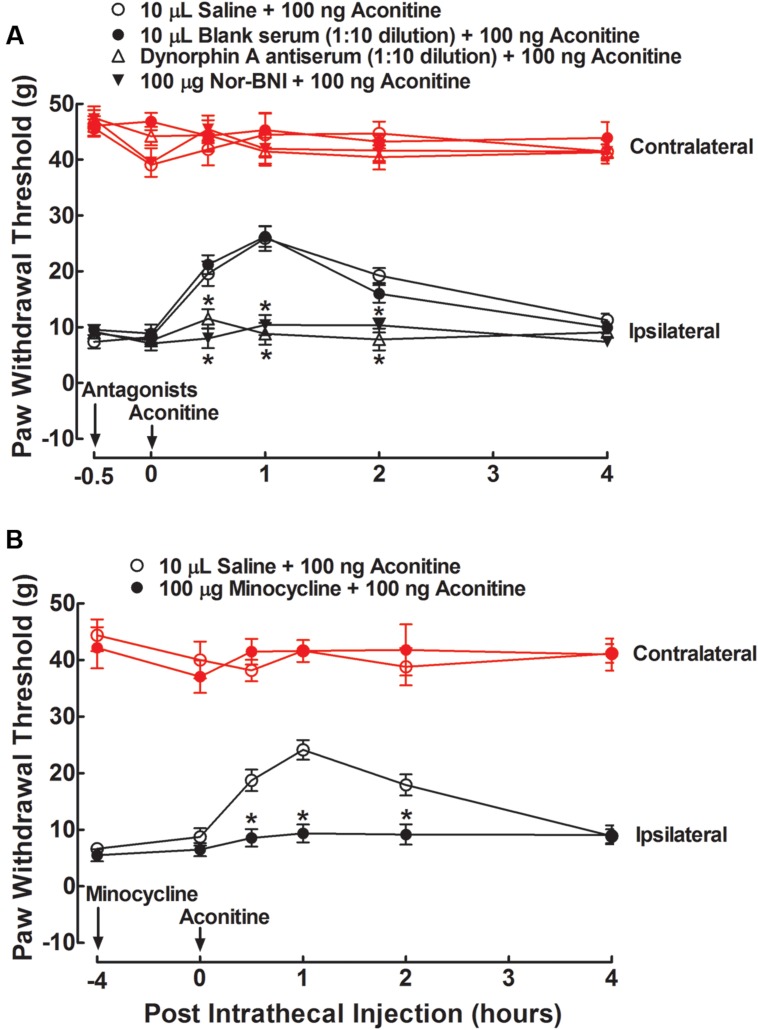
**Blockade effects of intrathecal injection of the specific dynorphin A antiserum **(A)**, selective κ-opioid receptor antagonist nor-BNI **(A)**, and microglial inhibitor minocycline **(B)** on spinal aconitine anti-allodynia in spinal L5/L6 nerve–ligated neuropathic rats.** The dynorphin A antiserum (1:10) and nor-BNI (100 μg) were given by intrathecal injection 0.5 h earlier than the intrathecal aconitine (100 ng) treatment, whereas minocycline (100 μg) was given 4 h earlier. Withdrawal responses to mechanical stimuli were measured in the contralateral and ipsilateral hindpaws. Data are presented as the mean ± SEM (six per group). ^∗^Denotes statistical significance compared with the saline + aconitine group (*P* < 0.05 by two-way repeated-measures ANOVA followed by the *post hoc* Student–Newman–Keuls test).

To test whether aconitine produced anti-allodynia through dynorphin A expression in the spinal cord, the specific dynorphin A antiserum (Fan et al., 2015) and selective κ-opioid receptor antagonist nor-BNI (Beardsley et al., 2010) were applied. Four groups of spinal nerve–ligated neuropathic rats received a single intrathecal injection of 10 μL saline solution, 10 μL blank rabbit serum (1:10 dilution), 10 μL dynorphin A antiserum (1:10 dilution), or nor-BNI (100 μg). A 100-ng dose of aconitine was given by intrathecal injection 0.5 h after the first injection. The dose and time regimens of the dynorphin A antiserum and nor-BNI were selected based on previous reports (Fan et al., 2015; Fan et al., 2016; Li et al., 2016). The paw withdrawal responses to mechanical stimuli were measured before, and 0.5, 1, 2, and 4 h after the second administration. As shown in **Figure 6A**, intrathecal injection of aconitine produced a marked and reversible anti-allodynic effect in the ipsilateral paws. Although intrathecal injection of the antiserum and nor-BNI did not affect the withdrawal responses in either the contralateral or ipsilateral paws as compared to the saline solution control, they completely blocked aconitine-induced anti-allodynia in the ipsilateral paws (*P* < 0.05 by two-way ANOVA followed by the *post hoc* Student–Newman–Keuls test).

To test whether aconitine produced anti-allodynia by means of spinal microglia, the microglial inhibitor minocycline (Raghavendra et al., 2003; Hua et al., 2005) was applied. Two groups of neuropathic rats received two intrathecal injections of saline solution (10 μL) + aconitine (100 ng), and minocycline (100 μg) + aconitine (100 ng), respectively. The second treatment was administered 4 h after the first treatment. The dose and time regimens of minocycline were selected according to the previous papers (Gong et al., 2014; Fan et al., 2015, 2016). The paw withdrawal thresholds for the response to mechanical stimuli were measured before, and 0.5, 1, 2, or 4 h after the second treatment. Intrathecal injection of minocycline did not affect withdrawal thresholds in either the contralateral or ipsilateral paws compared with the saline control. In the ipsilateral paws, a single intrathecal injection of aconitine led to marked and reversible anti-allodynia, which was completely prevented by pretreatment with minocycline (*P* < 0.05 by two-way ANOVA followed by the *post hoc* Student–Newman–Keuls test) (**Figure 6B**).

**FIGURE 7:**
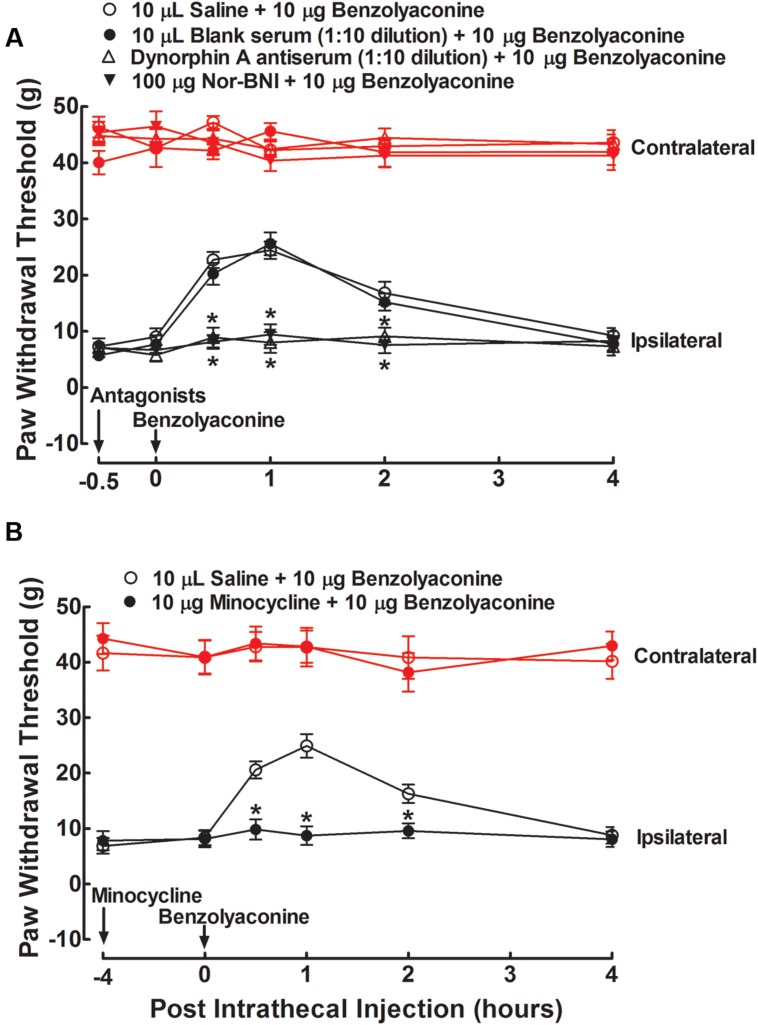
**Blockade effects of intrathecal injection of the specific dynorphin A antiserum **(A)**, selective κ-opioid receptor antagonist nor-BNI **(A)**, and microglial inhibitor minocycline **(B)** on spinal benzolyaconine anti-allodynia in spinal L5/L6 nerve–ligated neuropathic rats.** The dynorphin A antiserum (1:10) and nor-BNI (100 μg) were given by intrathecal injection 0.5 h earlier than the intrathecal benzolyaconine (10 μg) treatment, whereas minocycline (100 μg) was given 4 h earlier. Withdrawal responses to mechanical stimuli were measured in the contralateral and ipsilateral hindpaws. Data are presented as the mean ± SEM (six per group). ^∗^Denotes statistical significance compared with the saline + benzolyaconine group (*P* < 0.05 by two-way repeated-measures ANOVA followed by the *post hoc* Student–Newman–Keuls test).

To test whether the stimulatory effect of benzolyaconine on spinal dynorphin A expression was also causally associated with its anti-allodynia, the same treatment regimens of dynorphin A antiserum, nor-BNI, and minocycline on benzolyaconine were applied in six groups of neuropathic rats in the two separate studies. As shown in **Figure 7**, intrathecal injection of benzolyaconine (10 μg) produced marked and reversible anti-allodynia in the ipsilateral paws. Pretreatment with the dynorphin A antiserum (**Figure 7A**), nor-BNI (**Figure 7A**), and minocycline (**Figure 7B**) completely alleviated benzolyaconine anti-allodynia (*P* < 0.05 by two-way ANOVA followed by the *post hoc* Student–Newman–Keuls test).

### Stimulatory Effects of Aconitine, Benzolyaconine, and Aconine on the Prodynorphin Gene Expression in Cultured Primary Spinal Microglia

**FIGURE 8:**
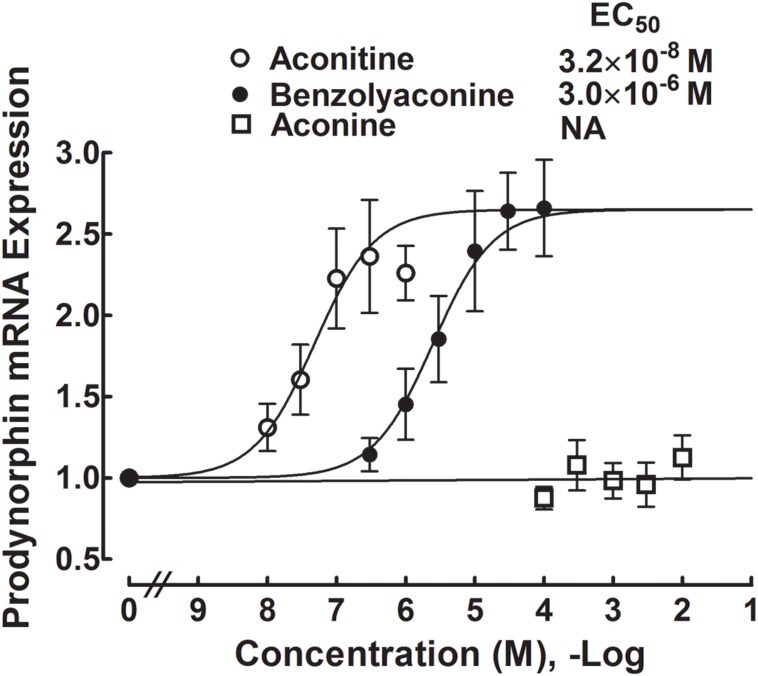
**Concentration-stimulatory response curves of aconitine, benzolyaconine, and aconine on the prodynorphin gene expression in primary cultures of spinal microglia.** Microglial cells originating from the spinal cords of the neonatal (1-day) rats were collected 2 h after incubation with the test articles. The expression of the prodynorphin gene (encoding dynorphin A) referred to the *gapdh* gene was measured with real-time quantitative PCR. The concentration-response analyses were best projected by the non-linear least-squares method. Data are presented as the mean ± SEM (three per treatment with two repeats).

Primary cultures of microglial cells taken from the spinal cords of the neonatal rats were treated with a variety of concentrations of aconitine (1 × 10^-8^, 3 × 10^-8^, 1 × 10^-7^, 3 × 10^-7^, and 1 × 10^-6^ M), benzolyaconine (3 × 10^-7^, 1 × 10^-6^, 3 × 10^-6^, 1 × 10^-5^, 3 × 10^-5^, and 1 × 10^-4^ M) and aconine (1 × 10^-4^, 3 × 10^-4^, 1 × 10^-3^, 3 × 10^-3^, and 1 × 10^-2^ M) for 2 h. As the bulleyaconitine A-increased dynorphin A level measured by the enzyme-linked immunosorbent assay was in parallel with the prodynorphin gene expression in spinal microglia from neonatal and adult rats (Li et al., 2016), the expression of the cellular prodynorphin gene encoding dynorphin A was only measured in this study using real-time quantitative PCR. As shown in **Figure 8**, both aconitine and benzolyaconine concentration-dependently increased prodynorphin gene expression, with the EC_50_ values of 32 nM and 3 μM, respectively. The EC_50_ ratio for aconitine and benzolyaconine was 1:94. However, treatment with aconine up to 10 mM did not significantly affect prodynorphin gene expression.

### Acute Spinal Neurotoxicity of Aconitine, Benzolyaconine, and Aconine

**FIGURE 9:**
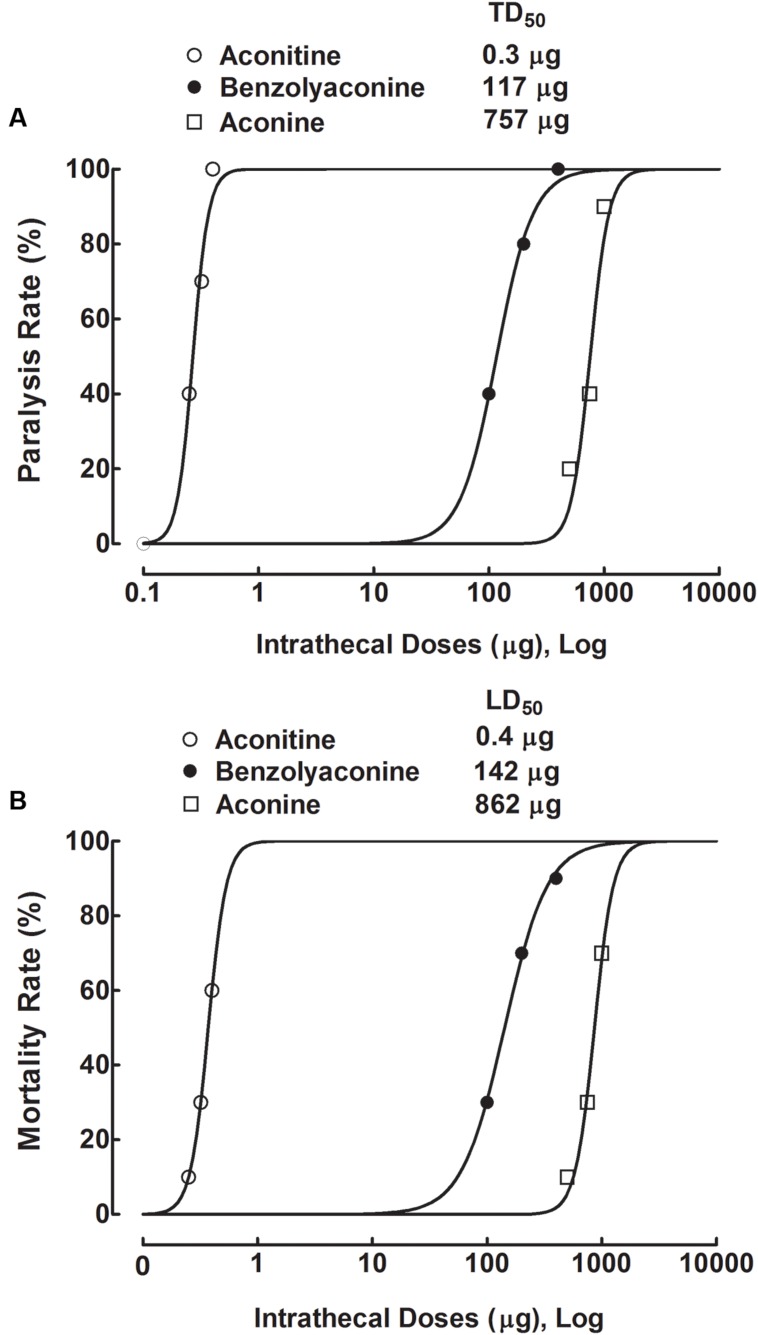
**Dose-response curves of intrathecal administration of aconitine, benzolyaconine, and aconine on flaccid paralysis **(A)** and death **(B)** in spinal L5/L6 nerve–ligated neuropathic rats.** Rats received a single intrathecal bolus injection of aconitine (0.25, 0.32, and 0.4 μg), benzolyaconine (100, 200, and 400 μg), and aconine (500, 750, and 1,000 μg). Paralysis and survival were observed for 24 h, with special attention given to the first 4 h. The dose-response analyses were best projected by the non-linear least-squares method. Data are presented as the percentage (10 per group).

To test the acute spinal neurotoxicity of aconitine, four groups of spinal nerve–ligated neuropathic rats received a single intrathecal bolus injection of aconitine (0.25, 0.32, and 0.4 μg). Abnormal behavior and survival were immediately observed for 24 h, with special attention given to the first 4 h. Intrathecal injection of aconitine evoked a range of abnormal behavior related to motor blockade, including cyanosis, asthma, abdominal breathing, locomotion difficulty, and flaccid paralysis in particular. The toxic behavior was immediate (a latency of 2–3 min), short-lasting (a duration of 5–10 min), and closely associated with death. As shown in **Figures 9A,B**, the flaccid paralysis and death were dose-dependent with a very steep shape in their dose-response curves. The dose-response analysis showed that the TD_50_ (for paralysis) and LD_50_ values were 0.3 μg or 0.4 nmol (95% confidence limits: 0.2–0.3 μg or 0.4–0.5 nmol) and 0.4 μg or 0.6 nmol (95% confidence limits: 0.4–0.4 μg or 0.6–0.6 nmol), respectively. However, the onsets of aconitine to induce paralysis and death were not dose-dependent, with paralysis latencies of 2.3 ± 0.7, 2.5 ± 0.8, and 2.6 ± 1.2 min and death latencies of 13.6, 12.4 ± 2.3, and 10.0 ± 3.3 min at 0.25, 0.32, and 0.4 μg, respectively.

Three groups of neuropathic rats received a single intrathecal bolus injection of benzolyaconine (100, 200, and 400 μg). Intrathecal injection of benzolyaconine caused immediate (2–3 min) and short-lasting (5–10 min) motor-blockade behavior, including flaccid paralysis and associated death (**Figures 9A,B**). The dose-response curve of benzolyaconine was less steep than that of aconitine. The dose-response analysis showed that the TD_50_ (for paralysis) and LD_50_ values were 117 μg or 0.2 μmol (95% confidence limits: 101–135 μg or 0.2–0.2 μmol) and 142 μg or 0.2 μmol (95% confidence limits: 130–156 μg or 0.2–0.3 μmol), respectively. The onsets of benzolyaconine to induce paralysis and death were not dose-dependent, and the latency values were similar to those of aconitine.

Three groups of neuropathic rats received a single intrathecal bolus injection of aconine (500, 750, and 1,000 μg). Intrathecal injection of aconine caused immediate and short-lasting motor-blockade behavior, including flaccid paralysis and associated death (**Figures 9A,B**). Analysis of the steep dose-response curves showed that the TD_50_ (for paralysis) and LD_50_ values were 757 μg or 1.5 μmol (95% confidence limits: 562–1,021 μg or 1.1–2.0 μmol) and 862 μg or 1.7 μmol (95% confidence limits: 779–954 μg or 1.6–1.9 μmol), respectively. The onsets of aconine at each dose were also independent of dose, with values similar to those of aconitine and benzolyaconine.

The TD_50_ (for paralysis) and LD_50_ values of aconitine, benzolyaconine, and aconine in mole base are presented in **Table 1**. Because death was closely associated with paralysis, the LD_50_ and TD_50_ values for paralysis were averaged to obtain a normalized TD_50_. The analysis showed that the TD_50_ (for paralysis) and LD_50_ ratios for aconitine vs. benzolyaconine were 1:452 and 1:421, with a normalized TD_50_ ratio of 1:400. For benzolyaconine vs. aconine, the TD_50_ (for paralysis) and LD_50_ ratios were 1:8 and 1:7, with a normalized TD_50_ ratio of 1:8. For aconitine vs. aconine, the TD_50_ (for paralysis) and LD_50_ ratios were 1:3750 and 1:2833, with a normalized TD_50_ ratio of 1:3200. The therapeutic index values of aconitine and benzolyaconine were also calculated. For aconitine, the TD_50_/ED_50_ (for paralysis) and LD_50_/ED_50_ ratios were 10:1 and 19:1, with a normalized TD_50_/ED_50_ ratio of 14:1. For benzolyaconine, the TD_50_/ED_50_ (for paralysis) and LD_50_/ED_50_ ratios were 56:1 and 57:1, with a normalized TD_50_/ED_50_ ratio of 56:1.

## Discussion

We recently made a conceptual advancement and identified that spinal microglial expression of dynorphin A is responsible for bulleyaconitine A anti-hypersensitivity in animal models of pain hypersensitivity, including formalin-induced tonic pain, neuropathic pain, and bone cancer pain (Li et al., 2016). In addition, Bullatine A, a C_20_-diterpenoid alkaloid, specifically stimulated dynorphin A expression in microglia in the spinal cord and cultured primary microglia (Huang et al., 2016). Dynorphin A is produced in many different parts of the brain, including the hypothalamus, striatum, and hippocampus, and in the spinal cord (Khachaturian et al., 1982; Smith and Lee, 1988; Mollereau et al., 1999). The expression and secretion of dynorphin A have been found in neurons (Minokadeh et al., 2010), astrocytes (Wahlert et al., 2013), and microglia (Li et al., 2016). Dynorphin A is an endogenous opioid neurotransmitter that modulates analgesic responses primarily by activation of the κ-opioid receptor located on neurons in the descending inhibitory system, although it has some affinity for μ- and δ-opioid receptors, and *N*-methyl-D-aspartic acid receptors (Nahin et al., 1989; Faden, 1990; Vanderah et al., 1996; Schwei et al., 1999; Rojewska et al., 2014). Our study further confirmed the causal relationship of aconitine between dynorphin A expression and antinociception, and suggest that the action of aconitine-evoked dynorphin A is via the activation of neuronal κ-opioid receptors. (1) Intrathecal injection of aconitine and its hydrolyzed metabolite benzoylaconine, but not aconine, markedly attenuated mechanical allodynia and heat hyperalgesia in a rat model of neuropathy induced by tight ligation of the spinal nerves. (2) The anti-allodynic effects of spinal aconitine and benzoylaconine were completely prevented by pretreatment with the specific dynorphin A antiserum, selective κ-opioid receptor antagonist nor-BNI, and microglial inhibitor minocycline. Minocycline has been extensively used to study the role of microglia in experimental models of brain ischemia (Yrjanheikki et al., 1999), traumatic brain injury (Sanchez Mejia et al., 2001), and peripheral nerve injury-induced neuropathy (Gong et al., 2014; Fan et al., 2015, 2016; Li et al., 2016). Minocycline is generally considered to be a specific inhibitor of microglia [but not astrocytes (Wu et al., 2002; Yoon et al., 2012) or oligodendroglial progenitors (Zhang et al., 2003)], but it was reported to have some direct biological effects in neurons (Wilkins et al., 2004; Huang et al., 2010). Although we also demonstrated that aconitine and benzoylaconine stimulated the prodynorphin gene expression in cultured primary microglial cells, studies are needed to provide more valid *in vivo* evidence of the microglial origin of dynorphin A, by applications of additional chemicals (such as liposome clodronate or PLS3397) and genetical manipulation. (3) Treatment with aconitine and benzoylaconine, but not aconine, significantly stimulated the expression of prodynorphin gene (encoding dynorphin A) in primary cultures of spinal microglial cells. In addition, we have previously shown that the aconitine analog bulleyaconitine A also stimulated the prodynorphin gene expression and increased the dynorphin A level in cultured primary microglial cells (but not astrocytes or neurons) from neonatal and adult rats, and the spinal cord of neuropathic rats *in vivo*, which was totally inhibited by pretreatment with minocycline (Li et al., 2016). Furthermore, the anti-hypersensitivity effects of the processed *Aconitum* extracts were also mediated by the stimulation of dynorphin A release and the activation of spinal κ-opioid receptors in rodent models of chronic constriction injury and repeated cold stress (Omiya et al., 1999; Suzuki et al., 1999; Xu et al., 2006). These results suggest that *Aconitum* and its contained diester diterpenoid aconitines cause anti-hypersensitivity primarily by stimulating the spinal microglial expression of dynorphin A, which passes the microglial neuronal synapses to activate κ-opioid receptors located on postsynaptic neurons.

The activity of aconitine on the stimulation of microglial dynorphin A expression (EC_50_ of 32 nM) appears to be more potent than bulleyaconitine A (EC_50_ of 45 nM) by approximately 0.5-fold. The greater potency of aconitine is also supported by the lower ED_50_ values of 39 and 32 pmol for intrathecal aconitine to block mechanical allodynia and heat hyperalgesia, which were approximately three to four times less than those (180 and 201 pmol) of bulleyaconitine A, respectively (Li et al., 2016). Being the C19-diterpenoid alkaloids, aconitine and bulleyaconitine A share similar structures, only with the major difference of two hydroxyl groups being attached at the C3 and C15 of aconitine (Li et al., 2016). This suggests that hydroxylation helps aconitines to increase the expression of spinal microglial dynorphin A and subsequent antinociception. The findings are consistent with the previous findings in acute pain. Yunaconitine has a C3-hydroxyl group, whereas bulleyaconitine A does not, and the C3-hydroxyl group of 3-acetylaconitine is acetylated. Systemic yunaconitine produced greater antinociception than bulleyaconitine A and 3-acetylaconitine in acetic acid and formalin tests (Yu et al., 1996).

Intrathecal benzolyaconine was able to block mechanical allodynia and heat hyperalgesia in neuropathy in a dose-dependent manner, but their normalized ED_50_ value (3.6 nmol) was 102 times greater than that of aconitine (35 pmol). More specifically, treatment with benzolyaconine increased the dynorphin A expression in cultured primary spinal microglial cells with an EC_50_ of 3 μM, which was also 94 times greater than that of aconitine (32 nM). These results indicate that the C8-acetylester group accounts for the effects of aconitine on the spinal microglial dynorphin A production and antinociception by the same degrees, which further support the concept that spinal microglial dynorphin A expression mediates aconitine-induced antinociception. The lesser potency of benzolyaconine in antinociception is also supported by the indirect evidence from other laboratories. It has been reported that benzoylaconine, benzoylmesaconine, and benzoylhypaconine inhibited acetic acid-induced writhing responses, with activities of around 100 times less than those of their parent compounds aconitine, mesaconitine, and hypaconitine (Hikino et al., 1979). It was also reported that the ED_50_ values for subcutaneous 3,15-diacetylbenzoylaconine (with the acetyl groups at both the C3 and C15) antinociception in the acetic acid, electric shock, and hot-plat assays were 12–16 times greater than those of 3-acetylaconitine (with the acetyl groups at both the C3 and C8) in mice and 25–30 times greater in rats (Zheng et al., 1994).

Intrathecal administration of aconitine produced acute neurotoxicity characterized by motor block-related flaccid paralysis and death, with a normalized TD_50_ of 0.5 nmol and a narrow therapeutic index (TD_50_/ED_50_) of 14:1. In contrast, the normalized therapeutic index of benzoylaconine was 56:1, reflecting the fact that deacetylation of the precursor aconitine more effectively reduces neurotoxicity than antinociception by three fold. The differential effect of deacetylation on neurotoxicity is also supported by the indirect evidence that the therapeutic index values of 3,15-diacetylbenzoylaconine by subcutaneous and intracerebroventricular injection were 0.4- to 0.9-fold and three-fold higher, respectively, than those of 3-acetylaconitine (Zheng et al., 1994). These results suggest that benzoylaconines, as secondary plant metabolites, may be prodrug candidates for the development of analgesics, given that primary aconitines, including bulleyaconitine A, 3-acetylaconitine, yunaconitine, and lappaconitine, have already been used as analgesics in clinical practice. The differential effects of aconitine and benzoylaconine on antinociception and toxicity also provide a scientific rationale for the *Aconitum* processing, which aims principally to decompose the diester diterpenoid aconitines into the relatively less toxic monoester diterpenoid benzoylaconines (Gozuacik et al., 2003; Singhuber et al., 2009; Sun et al., 2009; Lu et al., 2010). It has been reported that steaming of *Aconitum* at 120°C for 10 and 120 min resulted in 90.4 and 99.8% reduction of aconitine, and enhancement of benzolyaconine by 4.5- and 7.9-fold, respectively. Baking of *Aconitum* at 150°C for 10 and 120 min produced similar results with less degree, by 85.7 and 95.9% decreases in aconitine and enhancement of benzolyaconine by 3.0- and 4.1-fold, respectively (Yang et al., 2014). The processed *Aconitum* contains a mixture of bioactive aconitines and benzoylaconines, both of which contribute to certain extent to its analgesia (Hikino et al., 1979). Thus, the benzoylaconine-increased TD_50_/ED_50_ makes the processed *Aconitum* a more favorable analgesic, which is further enriched by the *in vivo* hydrolysis in the body. It was reported that blood benzoylaconines were increased and aconitines were decreased after oral administration of the processed *Aconitum* due to acetylester hydrolysis by carboxylesterases located in the gastrointestinal tract, intestinal bacteria, blood, and liver (Zhang et al., 2015).

Treatment with aconine up to 10 mM did not significantly stimulate the dynorphin A expression, which is associated with the finding that intrathecal injection of aconine up to 300 μg did not alter mechanical allodynia or thermal hyperalgesia in neuropathic rats. In contrast, intrathecal aconine caused dose-dependent acute neurotoxicity, with a normalized TD_50_ of 810 μg (1.6 μmol), which was seven times greater than that of benzoylaconine (0.2 μmol). Our results suggest that the C14-benzoly group is also required for aconitines to stimulate spinal microglial dynorphin A expression and associated antinociception, although it is less needed for neurotoxicity. The differential effects of benzolyaconine and aconine on antinociception and neurotoxicity further support the hypothesis that the antinociception induced by aconitines can be separated from their toxicity, the latter of which is presumably caused by the interactions with neuronal sodium channels (Ameri, 1998; Chan, 2009) and other possible mechanisms. In contrast, during the processing of *Aconitum*, the first step is to deacetylate at C8, but the second step of the debenzoylation at C14 is also inevitably involved, particularly when the procedures are overdone, although the benzoyl group is much more resistant to hydrolysis than the acetyl group, probably due to the mechanism of acyl migrations (Lerosen and Smith, 1949). The processed *Aconitum* mostly includes aconitines, benzoylaconines, and aconines (Huang et al., 2007). It was reported that steaming of *Aconitum* for 10 and 120 min led to increase in the aconine content by 1.5- and 10.3-fold, respectively (Yang et al., 2014). As we have demonstrated that benzolyaconine is antinociceptive with a favorable profile, whereas aconine is not antinociceptive but has certain toxicity (in contrast to the well-assumed “non-toxic”), measures should be cautionally made to avoid the overprocessing of *Aconitum* and excess hydrolysis of benzolyaconine to form aconine.

## Author Contributions

Conceived and designed the experiments: Y-XW and T-FL; performed the experiments: T-FL and NG; analyzed the data: T-FL, NG, and Y-XW; and wrote the paper: T-FL and Y-XW.

## Conflict of Interest Statement

The authors declare that the research was conducted in the absence of any commercial or financial relationships that could be construed as a potential conflict of interest.

## Abbreviations

ANOVA: analysis of variance
E_max_: maximum effect
EC_50_: median effective concentration
ED_50_: median effective dose
gapdh: glyceraldehyde-3-phosphate dehydrogenase
LD_50_: median lethal dose
% MPE: % maximum possible effect
nor-BNI: nor-binaltorphimine dihydrochloride
OX42: microglia marker
PCR: polymerase chain reaction
TD_50_: median toxic dose
